# A Symmetric Bidirectional Soft Rotary Actuator with a Bendable Shaft Driven by Extension-Type Pneumatic Actuators

**DOI:** 10.3390/s26185978

**Published:** 2026-09-21

**Authors:** So Shimooka, Shunsuke Mori, Tetsushi Kamegawa

**Affiliations:** Faculty of Environmental, Life, Natural Science and Technology, Okayama University, Okayama 700-8530, Japan

**Keywords:** soft rotary actuator, symmetry of characteristics, two-way rotation, hysteresis, bendable rotational axis

## Abstract

**Highlights:**

- A soft rotary actuator (SB-SRA) achieves bidirectional rotational motion by arranging extension-type flexible pneumatic actuators in an opposed configuration, even when its flexible shaft is bent.
- The rotation angle and torque are symmetrical in both directions (up to 105° and 0.51 Nm), and static hysteresis adjustment is demonstrated by adjusting the pressures of the opposing actuators.
- The high flexibility and safety of the SB-SRA make it promising as a soft robotic joint for rehabilitation and welfare applications.

**Abstract:**

Soft actuators capable of flexible motion have been actively studied and developed. Soft actuators that can extend and bend have been developed; however, soft rotary actuators have rarely been reported. In our previous studies, a soft rotary actuator (SRA) that generates rotational motion by constraining extension-type soft actuators (EFPAs) in a spiral shape was developed. However, the SRA had the limitation of rotating only in one direction, either clockwise or counterclockwise. In this paper, we aim to develop a symmetric bidirectional soft rotary actuator (SB-SRA) that achieves comparable rotational performance in both clockwise and counterclockwise directions while maintaining the flexibility of polymeric materials, such as rubber, and allowing static hysteresis adjustment. In particular, arranging several EFPAs so that they do not interfere with each other during extension enables the SRA to generate both clockwise and counterclockwise rotational motion. Unlike previously reported unidirectional soft rotary actuators, and unlike rigid antagonistic bidirectional joints that require bearings and a dedicated brake, the proposed SB-SRA achieves symmetric bidirectional rotation with a bendable rotational axis and enables static hysteresis compensation using only the opposing actuators. The results show that the maximum rotation angle and torque of the SB-SRA are approximately 105° and 0.51 Nm, respectively, under an input pressure of 500 kPa. The maximum directional differences between clockwise and counterclockwise operation were 3.3% for the rotation angle and 2.4% for the torque, demonstrating approximately symmetric output characteristics. In addition, static hysteresis adjustment can be achieved by varying the supply pressures applied to the opposing EFPAs.

## 1. Introduction

In recent years, soft actuators, which are composed of flexible materials such as rubber, films, and shape memory alloys, and can deform compliantly according to external environments and target objects, have been actively researched and developed [1,2,3]. Compared with conventional rigid mechanisms, soft actuators offer high flexibility and safety, making them promising for applications in robots that interact with humans, rehabilitation devices, and medical and welfare equipment [4,5]. In particular, pneumatic soft actuators have attracted considerable attention as driving elements that generate various motions because they are lightweight, structurally simple, and can produce large displacements by utilizing the elastic deformation of soft materials. Many studies have reported pneumatic soft actuators that generate extension and bending motions [1,2,6]. Pneumatic artificial muscles (PAMs) have also been widely investigated as compliant actuators for soft robotic systems. Ohta et al. developed a lightweight soft robotic arm using McKibben-type pneumatic artificial muscles and inflatable sleeves [7], while Zou et al. proposed muscle-fiber-array-inspired pneumatic artificial muscles capable of multiple deformation modes, including elongation, bending, and spiraling motions [8]. Recent studies have also investigated model-based design approaches in which approximate nonlinear models are used to simultaneously consider force/torque, bending performance, and controllability in the design of soft pneumatic actuators [9]. In addition, nonlinear dynamic models incorporating data-driven parameter estimation have been proposed to improve the prediction of the large-deformation behavior of soft pneumatic actuators [10].

However, for applications to robotic joints and rotary mechanisms, soft actuators that can directly generate rotational motion are required. In previous studies, Fukushima et al. developed a soft actuator driven by a mechanism that converts linear motion generated by pressurization into rotational motion [11]. In another approach, rotational motion was achieved by generating twisting motion through the pneumatic expansion of two spiral chambers [12]. In soft actuators made of flexible materials, achieving stable rotational motion remains challenging. In particular, a limited number of studies have reported soft rotary actuators that can generate rotational motion with equivalent characteristics in both rotational directions.

In our previous study, a soft rotary actuator (SRA) was developed by constraining extension-type flexible pneumatic actuators (EFPA) in a spiral shape [13,14]. This soft actuator can convert the extension motion generated by pneumatic pressure into rotational motion. However, the conventional structure has a fundamental limitation in that it can rotate only in one direction, either clockwise or counterclockwise. In addition, hysteresis occurs between the pressurization and depressurization processes owing to the deformation characteristics of friction between the silicone rubber tube and bellows-type nylon sleeves. These issues reduce the controllability and versatility of the actuator when it is used as a practical rotary driving mechanism. These limitations indicate the need for a soft rotary actuator that can generate bidirectional rotational motion with comparable rotation-angle and torque characteristics in both directions, while retaining a flexible rotational axis and enabling hysteresis compensation without additional braking components.

Therefore, in this study, we aim to develop a soft rotary actuator that can rotate in both clockwise and counterclockwise directions and has symmetric output characteristics in both directions. In the proposed actuator, extension-type flexible pneumatic actuators that generate rotational motion in opposite directions are combined while bending the shaft. By pressurizing one actuator, rotational motion is generated in one direction, while the other actuator functions as the driving element for the opposite direction. This structure is designed to overcome the one-directional rotation limitation of the conventional actuator and to achieve equivalent rotation angles and torques in both clockwise and counterclockwise directions. To verify the symmetric characteristics in both directions, the rotation angle and torque of the SRA in both directions were measured. The results indicate that the characteristics of the SRA in both rotational directions are nearly identical.

Based on the structure of the SRA, we investigated whether the hysteresis in rotational motion between pressurization and depressurization could be reduced by adjusting the supply pressure applied to each EFPA. The results indicate that the hysteresis in rotational motion was reduced by adjusting the supply pressure applied to the other EFPA while maintaining a constant pressure in one EFPA. Therefore, this study realized a soft actuator that enables static compensation of the hysteresis in the rotation angle by simply adjusting the pressure applied to each EFPA in the SRA. Although the SRA undergoes slight deformation, the proposed actuator enables angle adjustment comparable to that of conventional rotary actuators [14] while allowing the rotational axis to bend.

The main contributions of this paper are summarized as follows:

(1) A new soft rotary actuator architecture that generates bidirectional rotational motion by arranging EFPAs so that each extends toward its corresponding rotational direction, with both directions transmitted through a single common output plate.
(2) Experimental verification of approximately symmetric rotation-angle and torque characteristics in both rotational directions, with maximum directional differences of 3.3% for the rotation angle and 2.4% for the torque over the tested pressure range.
(3) A static hysteresis compensation method uses only the opposing EFPAs, without requiring additional components such as a brake.
(4) Experimental demonstration of bidirectional rotation while maintaining a bendable rotation axis.

## 2. Previous Soft Rotary Actuator (SRA) and Related Work

### 2.1. Extension-Type Flexible Pneumatic Actuator (EFPA)

**Figure 1:**
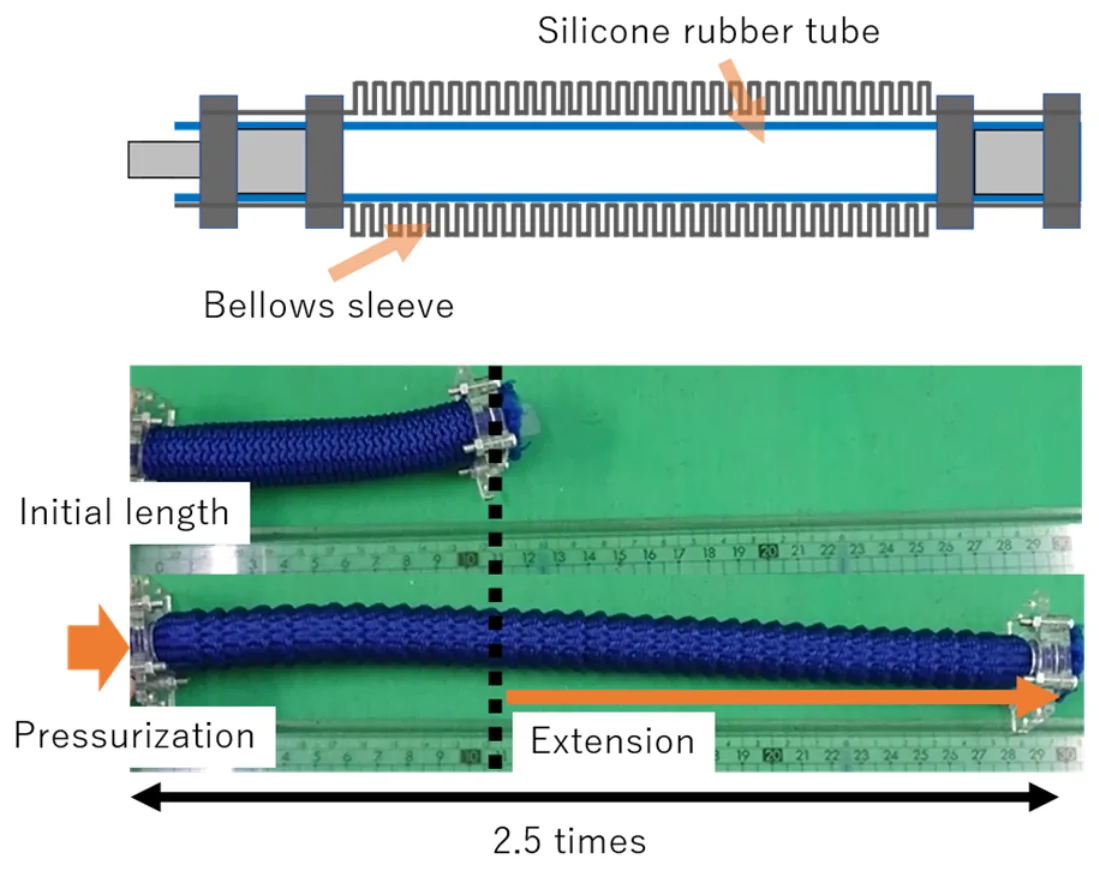
Construction and operation of EFPA.

As a soft actuator used to generate rotational motion, an extension-type flexible pneumatic actuator (EFPA) with an elongation ratio of approximately 2.5, as shown in Figure 1, was proposed [13]. The EFPA consists of a silicone rubber tube covered with a bellows sleeve. The inner diameter and wall thickness of the silicone rubber tube are 8 mm and 1.5 mm, respectively. The outer diameter of the bellows sleeve is 20 mm. These dimensions and flexible constituent materials allow the EFPA to generate axial extension even in a bent configuration. The operating principle of the EFPA is as follows. First, the silicone rubber tube expands in both the radial and longitudinal directions when pressurized. Because the bellows sleeve constrains radial expansion and allows deformation mainly in the axial direction, the EFPA extends longitudinally. As shown in Figure 1, the EFPA extends to approximately 2.5 times its initial length in the axial direction when the rubber tube is pressurized [13]. The EFPA offers high flexibility and can bend easily without damage even while extended. In addition to these characteristics, the EFPA maintains high flexibility during extension, which is an advantage over previous linear pneumatic actuators and other reported soft actuators [15,16,17,18,19,20,21].

### 2.2. Previous Soft Rotary Actuator with a Flexible Shaft

**Figure 2:**
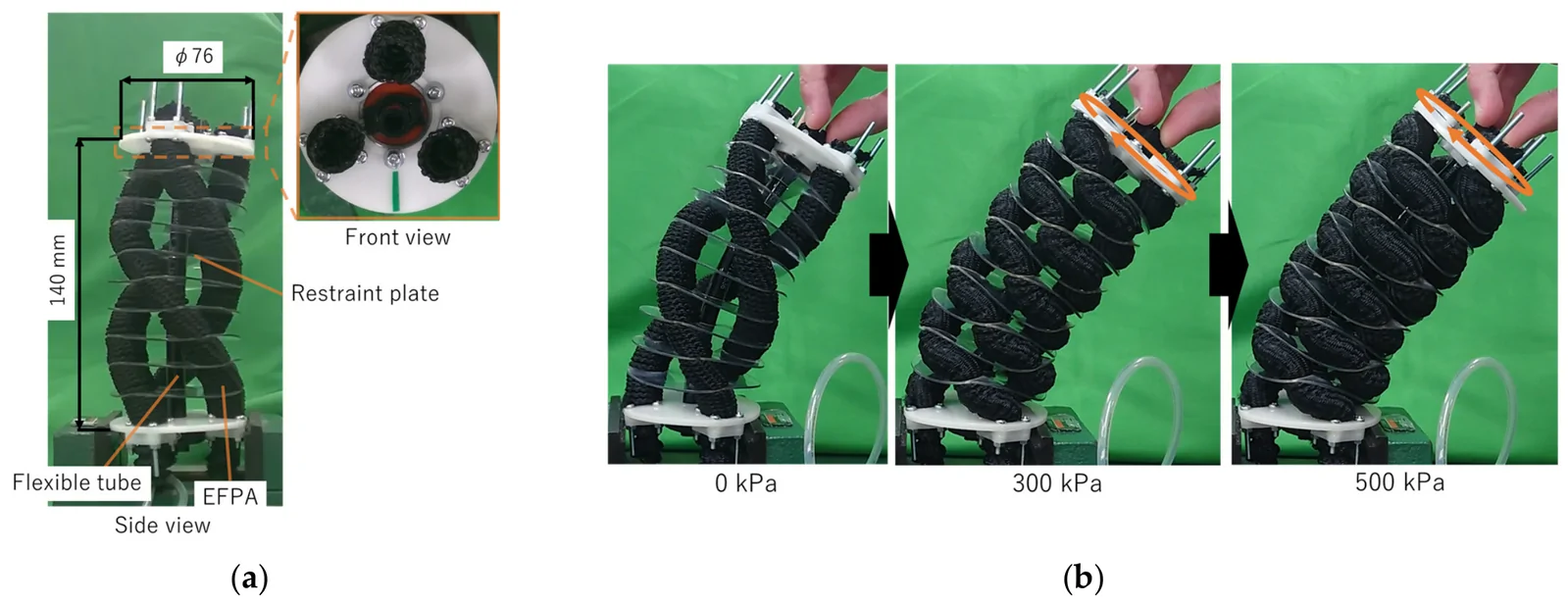
(**a**) Overview of SRA equipped with flexible shaft; (**b**) transient view of the SRA motion with the shaft bent during gradual pressurization.

Figure 2a shows an SRA equipped with a polyurethane tube with an outer diameter of 10 mm as the rotational shaft [14]. The SRA consists of a flexible tube shaft, three EFPAs, nine polyethylene terephthalate (PET) plates, and upper and lower end stages. Each helically arranged EFPA is inserted into holes in the PET plates. The three EFPAs are placed at intervals of 120° to avoid interference with each other. Each EFPA is restrained at every seven pitches by the nine PET plates, and both ends of the EFPAs are fixed to the upper and lower stages, as shown in Figure 2a. Inserting the bellows sleeve of each EFPA into the holes in the PET plates allows the distance between adjacent plates to be maintained even when the SRA is pressurized. In addition, the SRA can rotate without buckling because the three EFPAs are uniformly constrained by the PET plates. When pressurized, each EFPA extends along its longitudinal axis, resulting in rotational motion of the SRA. Owing to the helical arrangement of the EFPAs, their axial extension is converted into circumferential displacement, which generates rotational motion of the SRA. To demonstrate that the SRA can rotate while the flexible shaft is bent, Figure 2b shows transient views of the SRA motion when the shaft was bent at 45°, and the pressure was gradually increased. As evident from Figure 2b, the SRA can rotate even if the shaft is bent, and none of the EFPAs interfere with the motion of the others. Therefore, it can be described that the proposed SRA is a highly flexible rotary actuator.

However, the developed SRAs had difficulty achieving bidirectional rotational motion because the EFPAs extended in the same direction. Bidirectional rotational motion of the SRA can be achieved by controlling the pressurization of the EFPAs so that the SRA reaches its neutral position. The torque characteristics of the bidirectional motion are asymmetric because the driving mechanisms differ between the two rotational directions: one direction is driven by supply pressure, whereas the other is driven by the restoring force of the silicone rubber.

### 2.3. Comparison with Related Work

Soft torsional actuators such as vacuum-powered twisting actuators [22], origami-based twisting actuators [23], and low-profile soft rotary actuators [24] generate rotational motion in a specific direction and therefore cannot achieve bidirectional rotation with a single structure. Antagonistic actuation for bidirectional torsion was proposed by Sanan et al. [25] using fabric-based actuators without rigid components; however, this work explored the design concept, reported that such actuators are best suited to binary actuation between two discrete states, and did not provide experimental characteristics of the rotation angle, output torque, or symmetry. Unlike the bidirectional joint in [26], which consists of a rigid aluminum housing and a central shaft supported by bearings and requires a dedicated pneumatic brake and an antagonistic preload to maintain its position, the SRA requires neither bearings nor a dedicated brake and can rotate and maintain the rotation angle even when its flexible shaft is bent. In addition, the rotation angle can be maintained simply by adjusting the supply pressures of the opposing EFPAs.

Table 1 compares the proposed SB-SRA with representative soft rotary and torsional actuators reported in the literature; the values for the SB-SRA are those obtained in Section 4. The joint reported in [26] produces a larger torque of 4.76 Nm, but it weighs 1.5 kg and is 110 mm in diameter and 180 mm long because of its aluminum housing and ball bearings, whereas the SB-SRA weighs 0.5 kg with the same diameter and half the length. Although hysteresis of the pneumatic artificial muscles was observed in the reference [26], no compensation method was presented. In contrast, the SB-SRA compensates for the hysteresis by adjusting the pressures of the opposing EFPAs, without any additional components. To the best of our knowledge, the SB-SRA is the first rotary actuator that provides continuously adjustable symmetric bidirectional rotation, operates with a bent rotational axis, and maintains its angle without bearings or a dedicated brake.

Therefore, this study aims to develop an SRA that can generate symmetric bidirectional rotational motion with comparable characteristics of rotation angle and torque in both directions while retaining its advantages. In the proposed design, both rotational directions are transmitted through a single common output plate.

## 3. Design of a Symmetric Bidirectional Soft Rotary Actuator (SB-SRA)

### 3.1. Mechanism of SB-SRA

**Figure 3:**
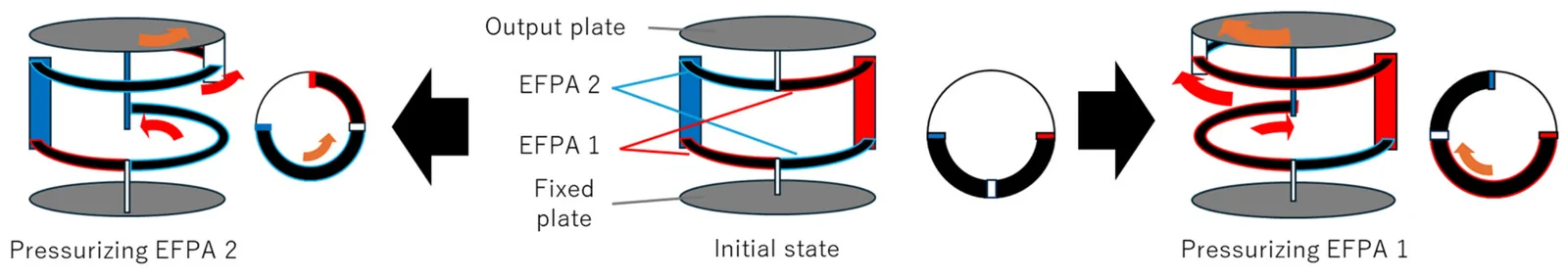
Operating principle of the SB-SRA for bidirectional rotational motion.

To address this issue, we aim to develop an SB-SRA that can generate bidirectional rotational motion and allow static hysteresis adjustment while retaining the ability to rotate when the flexible shaft is bent using the EFPA. Therefore, the arrangement of the EFPAs must be carefully designed to enable bidirectional rotational motion without mutual interference. Figure 3 shows the operating principle of the SB-SRA for bidirectional rotational motion. The EFPAs are arranged in diagonally opposed pairs, with one pair assigned to each rotational direction, allowing torque to be transmitted to the output plate in both the clockwise and counterclockwise directions. Therefore, the SB-SRA can rotate in either the clockwise or counterclockwise direction by selectively applying supply pressure to the corresponding EFPAs. The unpressurized EFPAs passively follow the motion of the output plate without hindering its rotation, enabling smooth rotational motion of the SB-SRA. Additionally, when EFPAs 1 and 2 are pressurized, the output torque of the SB-SRA can be adjusted by controlling the supply pressure, similar to conventional rotary actuators. Section 4 presents the characteristics of the SB-SRA under controlled internal pressures in EFPAs 1 and 2.

### 3.2. Rotational Motion and Characteristics of SB-SRA

**Figure 4:**
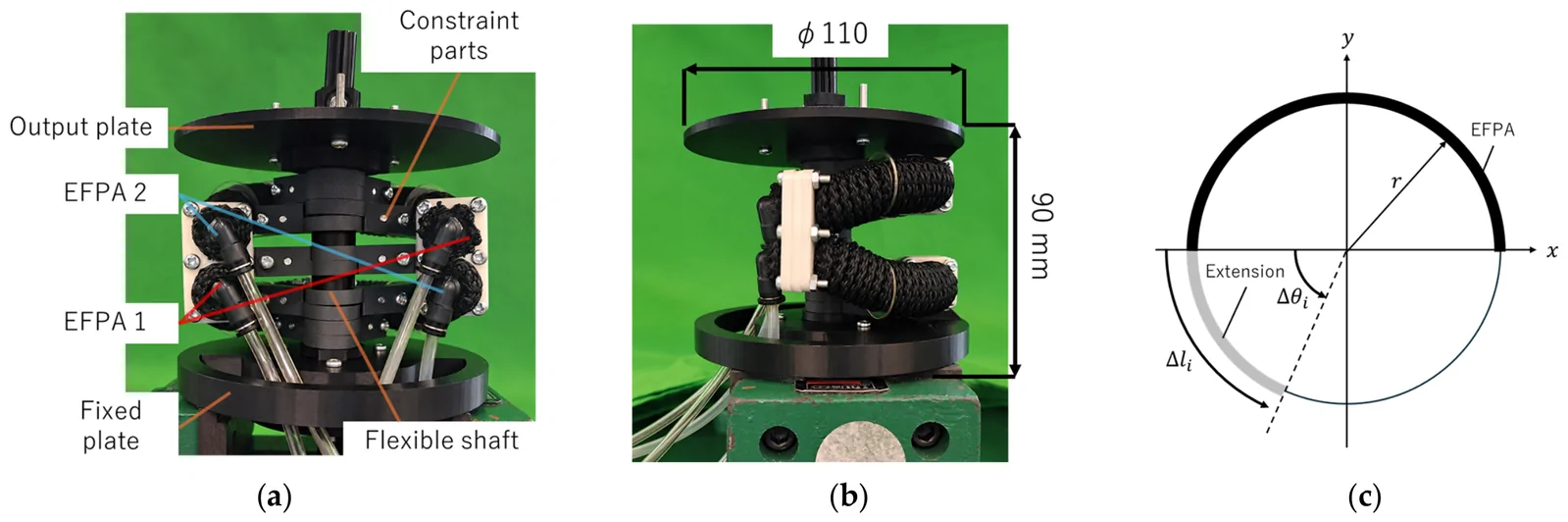
(**a**) Overview of SB-SRA from the front view; (**b**) from the side view of the SB-SRA; (**c**) schematic of the simplified rotation-angle model.

Figure 4 shows the overview of the SB-SRA. The SB-SRA, which is designed to achieve bidirectional rotational motion, consists of four EFPAs, an output plate, a fixed plate, a flexible shaft, and constraint parts, as shown above. As shown in Figure 4, the constraint parts maintain the prescribed posture of the EFPAs and guide their extension in the rotational direction, while the flexible shaft allows the rotation axis to bend.

To estimate the rotation angle of the SB-SRA, the extension direction of each EFPA was considered. Because each EFPA is arranged obliquely with respect to the circumferential direction, the extension displacement of the EFPA is not directly equal to the circumferential displacement of the output plate. Therefore, the circumferential component of the EFPA extension was used to estimate the rotation angle. As shown in Figure 4c, when the circumferential displacement induced by the extension of the corresponding EFPA is defined as $∆l_{i}$, the rotation angle of the SB-SRA ${∆\theta }_{i}$ generated in rotational direction i is simply expressed as

(1) $$∆\theta_{i}=\frac{{∆l}_{i}}{r},$$

where r is the distance (45 mm) from the rotation center to the EFPA (length: 65 mm). Here, i denotes the rotational direction, such as clockwise or counterclockwise (1=CW and 2=CCW). In this simplified model, changes in the three-dimensional posture of the flexible EFPA during rotation are not explicitly considered. To avoid introducing the changing inclination angle of the EFPA as an additional variable, the displacement induced by the EFPA extension is approximated as an in-plane circumferential displacement. Therefore, Equation (1) represents a simplified geometric relationship between the EFPA-induced circumferential displacement and the output rotation angle. Unlike nonlinear dynamic models that explicitly consider material properties and dynamic behavior, the present relation is intended as a simplified quasi-static geometric model for estimating the basic rotational behavior of the proposed mechanism.

The length of each EFPA in the SB-SRA is designed to achieve bidirectional rotational motion with a rotation angle of more than 90° in each direction.

## 4. Characteristics of SB-SRA

### 4.1. Rotation-Angle Characteristics of SB-SRA

**Figure 5:**
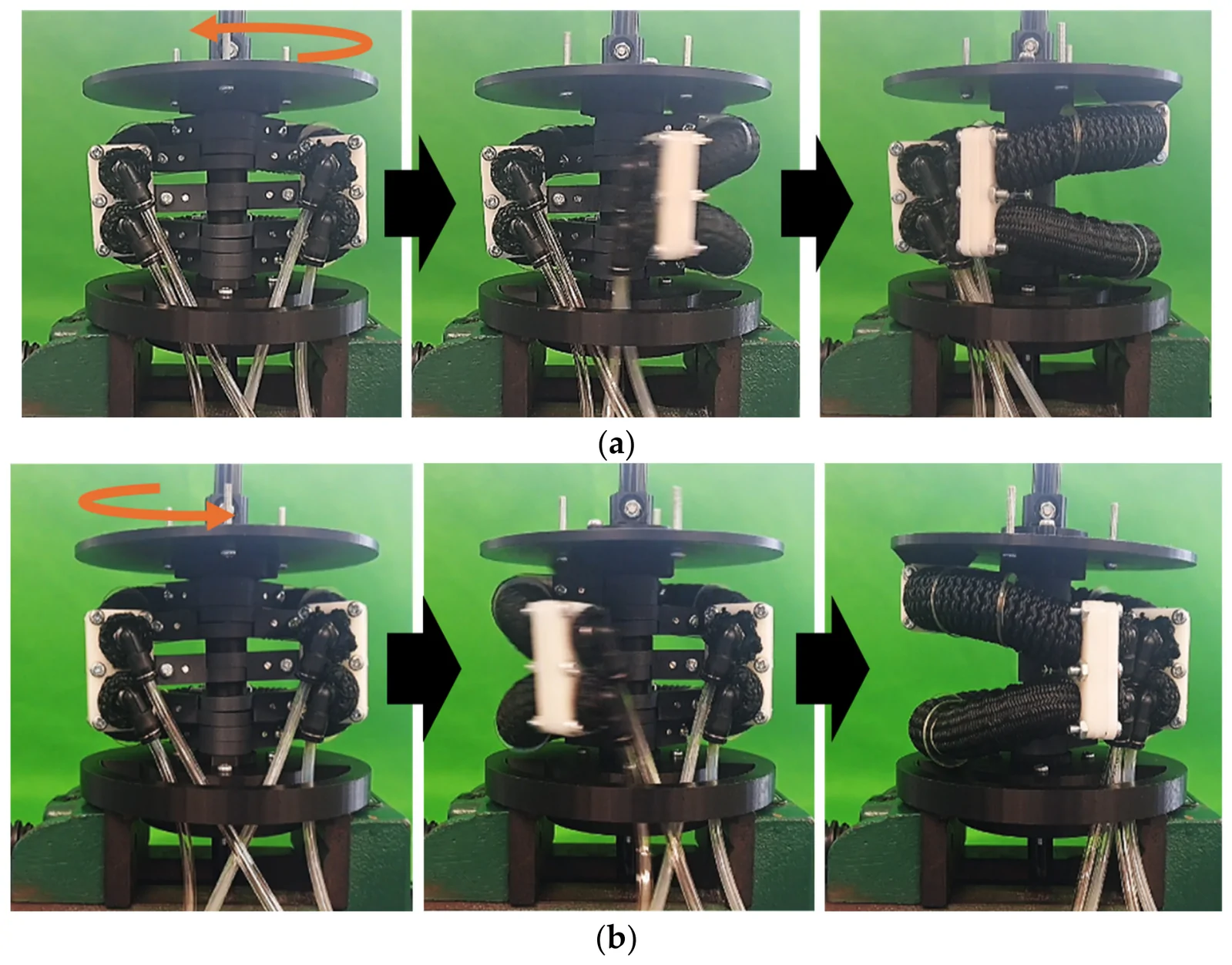
Transient views of the SB-SRA: (**a**) clockwise rotation; (**b**) counterclockwise rotation.

**Figure 6:**
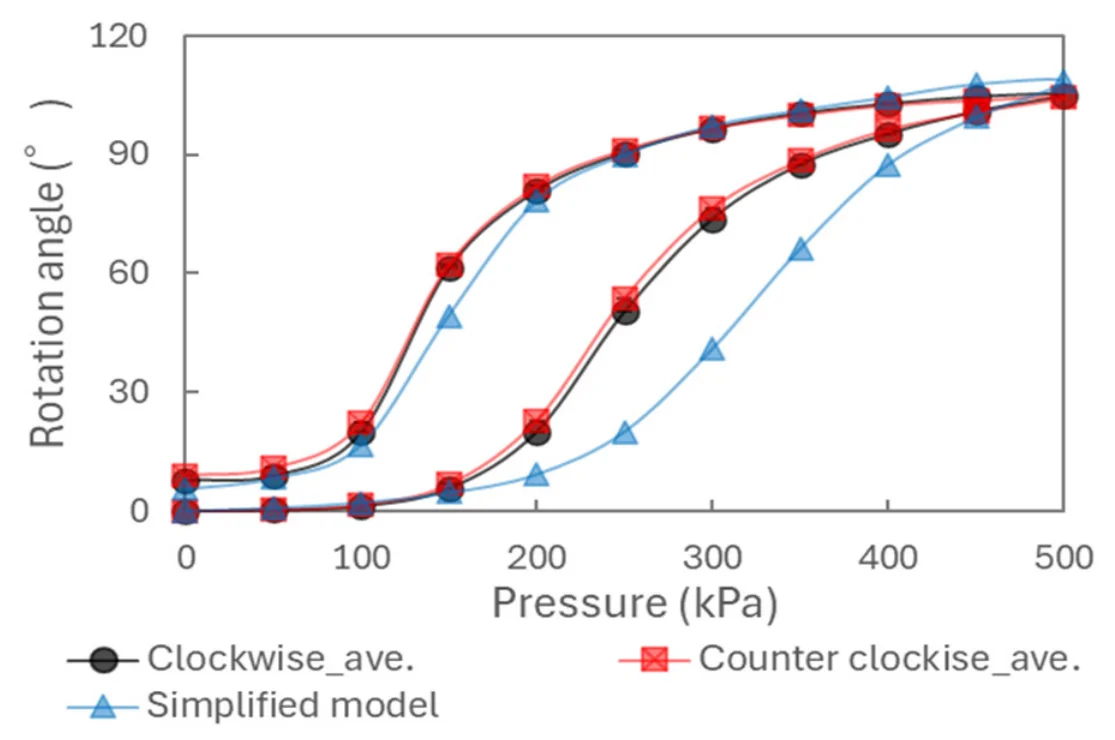
Relationship between pressure and the output rotation angle of the SB-SRA.

Figure 5 demonstrates the transient view of the bidirectional rotational motion of the SB-SRA. Figure 5a shows the transient views when it rotates clockwise, and Figure 5b shows the views when it rotates counterclockwise. Figure 5 shows that the SB-SRA achieves bidirectional rotational motion by selectively applying supply and exhaust pressure to the corresponding EFPAs. The supply pressure applied to each EFPA was monitored using a pressure sensor (KEYENCE, Osaka, Japan, AP-53A, resolution of 1 kPa). In the experiments, the rotation angle was measured three times in each rotational direction, and the torque was measured at four installation positions with three trials each. Figure 6 shows the relationship between the supply pressure and the output rotation angle of the SB-SRA. In the experiment, the rotation angles in both clockwise and counterclockwise directions were measured three times using image analysis. To compare the rotational characteristics in both directions, the absolute values of the rotation angles are plotted in Figure 6.

To evaluate the applicability of the simplified planar model expressed by Equation (1), the estimated rotation angle was compared with the measured rotation angle, as shown in Figure 6. The estimated values generally followed the trend of the experimental results, although the maximum error was 32.3°. The measured rotation angle increased more rapidly than the estimated value in some pressure ranges. This difference may be attributed to changes in the friction between the rubber tube and the bellows sleeve during the extension of the EFPAs.

The results show that the rotation angles in both directions were in good agreement, even when the EFPAs were decompressed. The maximum difference between the clockwise and counterclockwise rotation angles was 1.17°, and the maximum standard deviation for each supply pressure was 0.83°. To quantitatively evaluate the directional symmetry, the directional difference was calculated as the absolute difference between the mean clockwise and counterclockwise outputs divided by the corresponding maximum measured output at 500 kPa. The maximum directional difference in the rotation angle over the tested pressure range was 3.3% at a supply pressure of 250 kPa.

This result indicates that the proposed soft actuator can achieve bidirectional rotational motion with symmetrical rotational characteristics in both directions. The maximum rotation angle of approximately 105° was obtained at 500 kPa. This maximum angle is mainly limited by the initial length and achievable extension of the EFPAs, together with their geometric arrangement in the SB-SRA.

However, hysteresis was observed between pressurization and decompression, which was caused by friction between the bellows sleeve and the silicone rubber tube.

**Figure 7:**
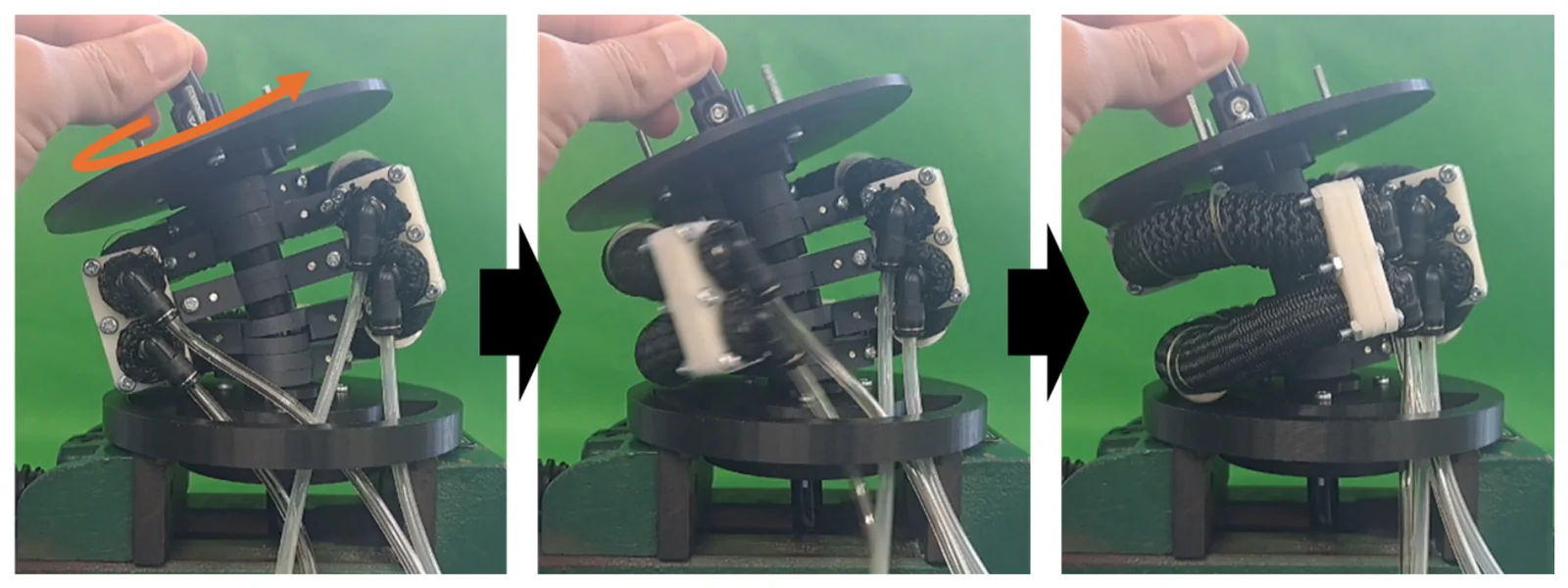
Rotational motion while bending the shaft.

In functional testing, the SB-SRA could generate bidirectional rotational motion with the flexible shaft bent at an angle of 20° as shown in Figure 7. These results demonstrate that the proposed SB-SRA can maintain bidirectional rotational motion while retaining a bendable rotational axis.

**Figure 8:**
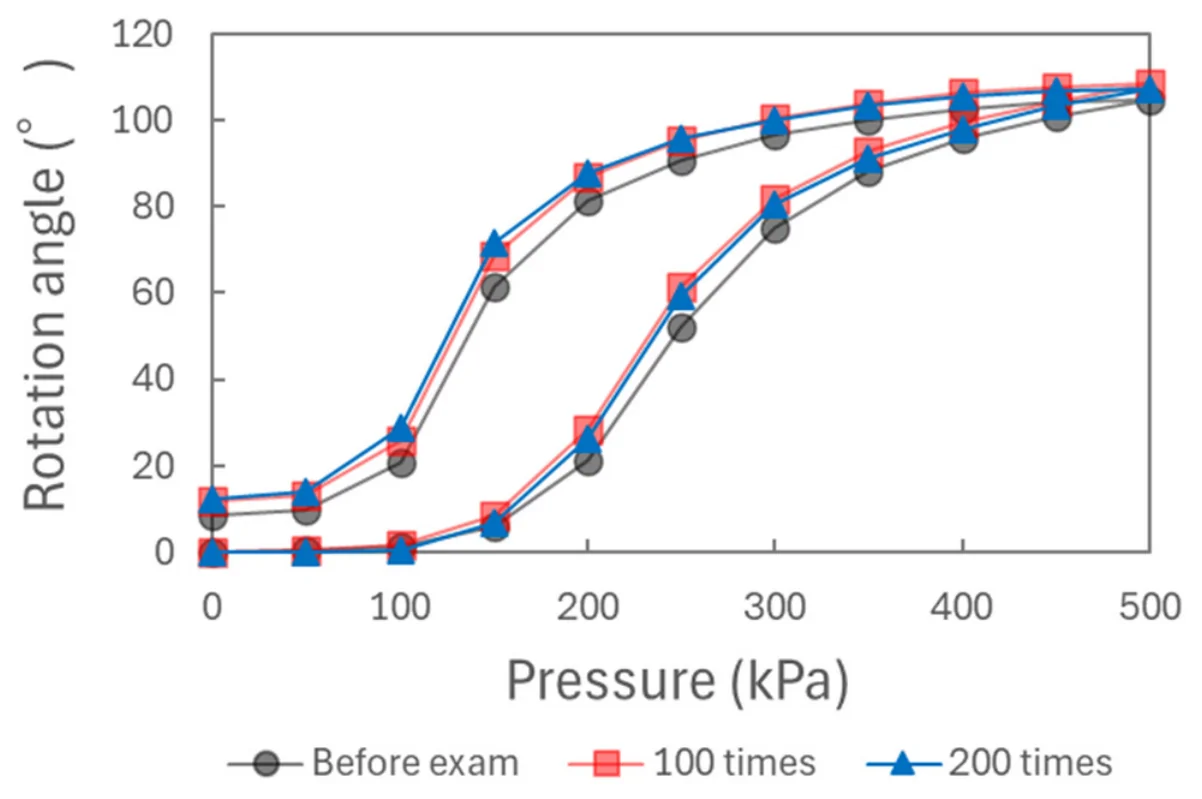
Relationship between the supply pressure and the rotation angle of the SB-SRA after 100 and 200 repeated pressurization cycles.

The rotation-angle characteristics of the SB-SRA were investigated after 100 and 200 repeated pressurization cycles from 0 to 500 kPa. Figure 8 shows the relationship between the supply pressure and the rotation angle of the SB-SRA after 100 and 200 repeated pressurization cycles.

The results showed that the rotation-angle characteristics shifted toward larger rotation angles after 100 cycles. However, the rotation-angle characteristics did not change significantly between 100 cycles and 200 cycles. This behavior may be caused by the elongation of the silicone rubber tube during repeated actuation, while the bellows sleeve constrains excessive extension of the EFPA.

**Figure 9:**
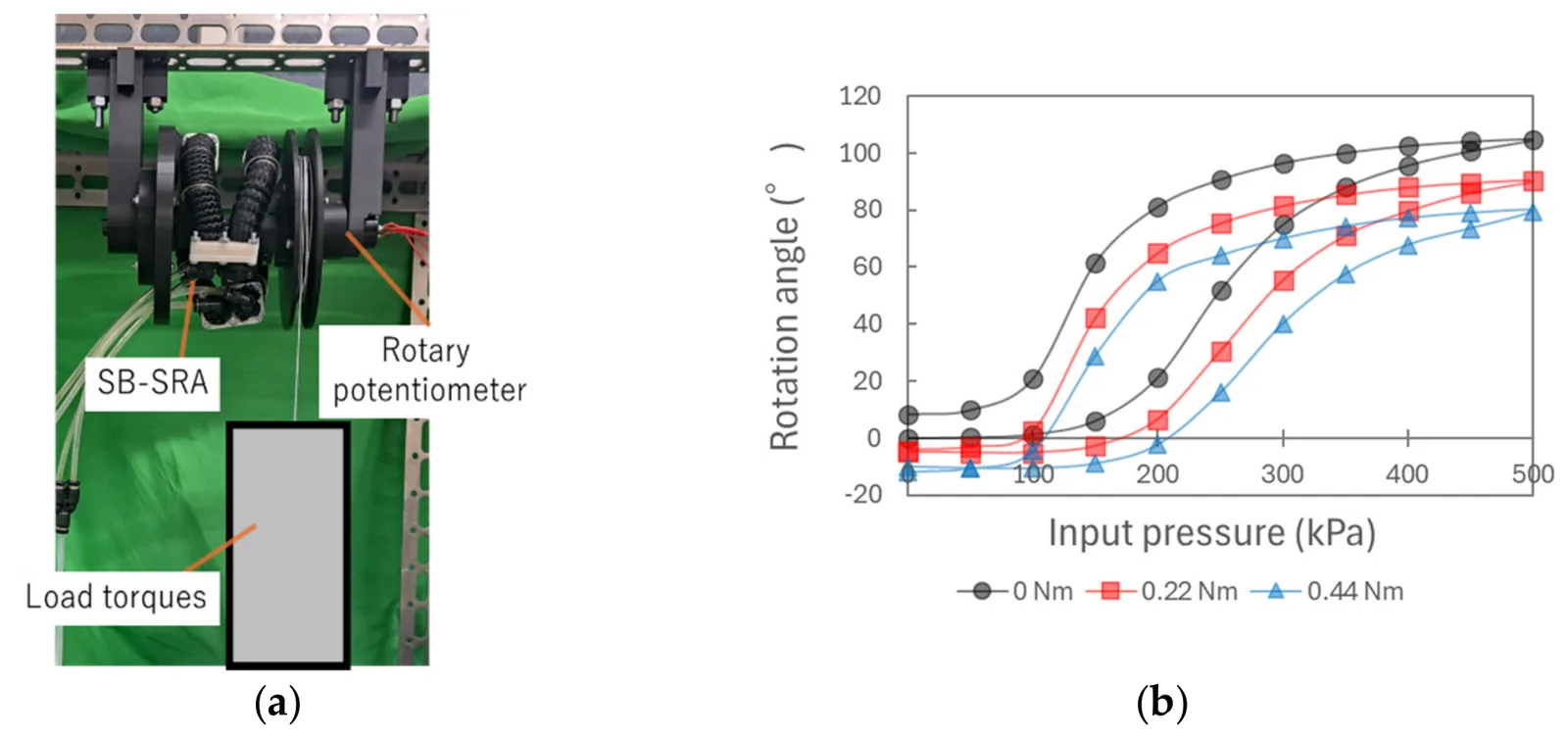
(**a**) Experimental setup to measure the rotation angle under different weights; (**b**) relationship between the supply pressure and the rotation angle of the SB-SRA under different weights.

Next, the influence of the load torque on the rotation-angle characteristics of the SB-SRA was investigated. In the experiment, the bottom plate of the SB-SRA was fixed to a stand, and the top plate, to which the load torques (0.22 Nm and 0.44 Nm) were applied via a wire and weights (0.5 kg and 1.0 kg), was connected to a rotary potentiometer (Midori Precisions, Tokyo, Japan, CP-2FB(b)J, 5 kΩ) as shown in Figure 9a. Figure 9b shows the relationship between the supply pressure and the rotation angle of the SB-SRA under different loads. It was found that the rotation angle decreased by approximately 12.6° for every 0.22 Nm of load torque.

### 4.2. Torque Characteristic of SB-SRA

**Figure 10:**
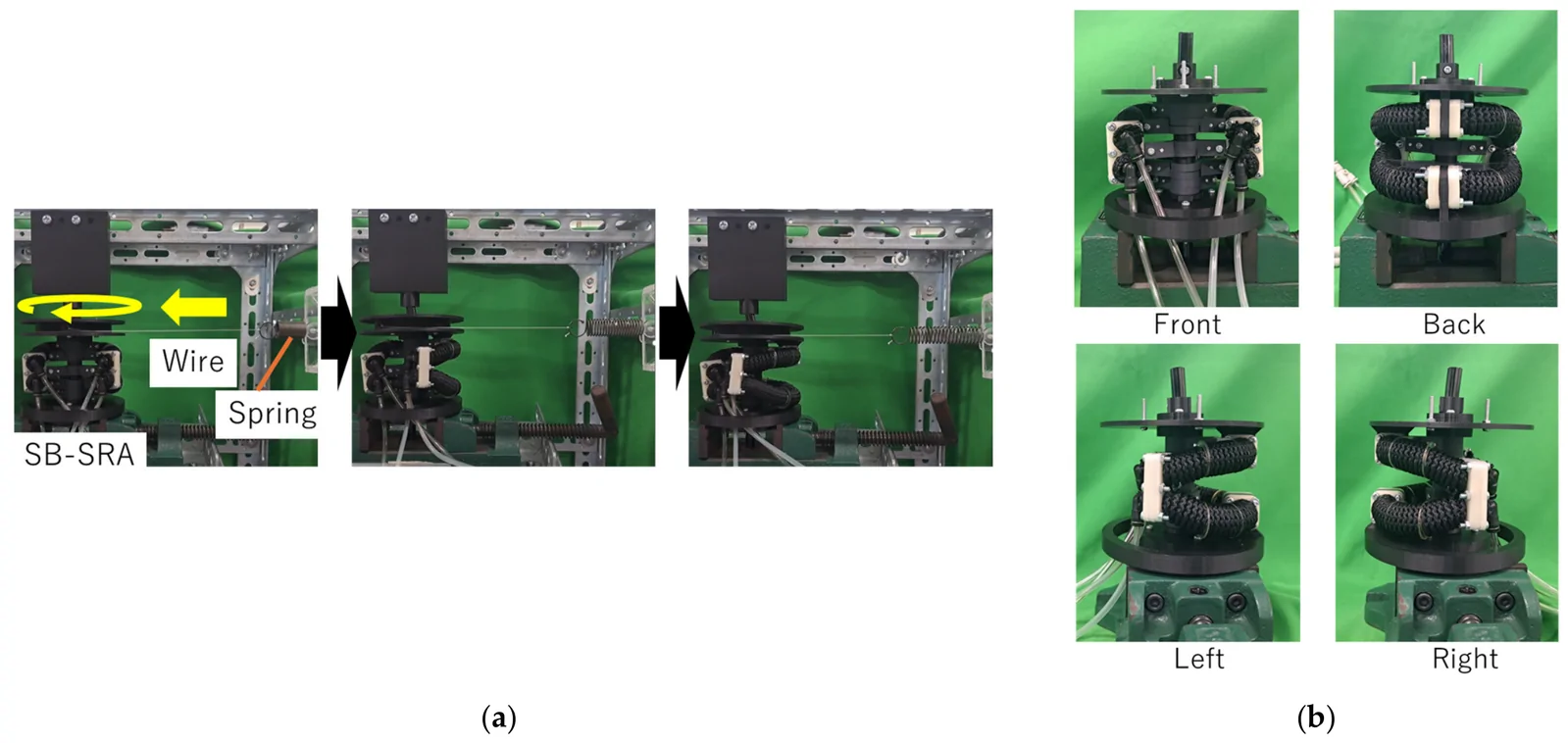
(**a**) Experimental setup to measure the torque by rotational motion; (**b**) positions of the SB-SRA for measurement.

**Figure 11:**
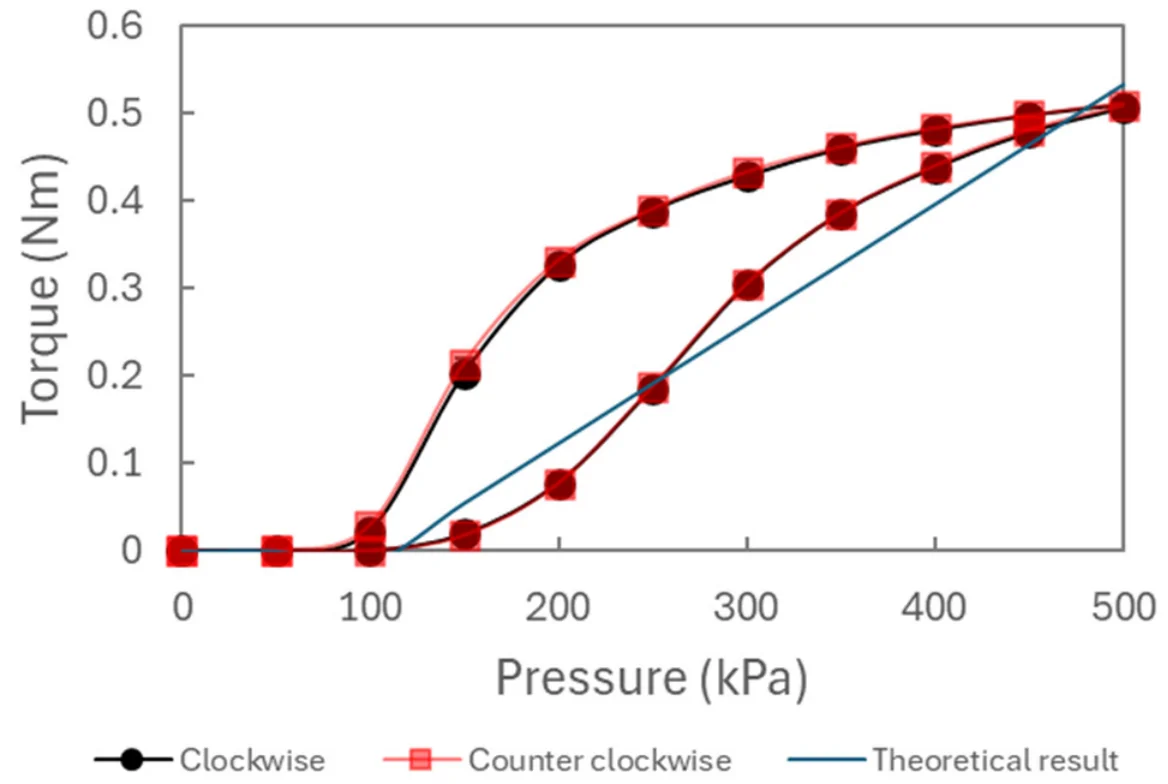
Relationship between pressure and torque of the SB-SRA.

Next, the torque characteristics of the SB-SRA were measured to evaluate their symmetry. Figure 10a shows the experimental setup to measure the torque by rotational motion. The upper end of the SRA is connected to a tensile spring (Accurate Inc., Saitama, Japan, DE660, spring constant 0.3 N/mm) as shown in Figure 10a. This measuring method is often used for a rotary actuator. The torque was calculated using the pulling force exerted on the spring when the SRA was pressurized and decompressed. In the experiment, the torque of the SB-SRA was measured three times for the four installation positions shown in Figure 10b, because the actuator configuration is not geometrically symmetric and the torque characteristics may vary depending on the installation position. Figure 11 shows the relationship between pressure and torque of the SB-SRA. The results show that the torque characteristics of the SB-SRA were approximately symmetrical, just like the rotation-angle characteristics shown in Figure 6. The maximum absolute difference between the mean clockwise and counterclockwise torques was 0.0122 Nm during decompression at 150 kPa, and the maximum standard deviation for all supply pressures was 0.0066 Nm. Using the same definition as that for the rotation angle, the maximum directional difference in torque was 2.4% during decompression at 150 kPa. The torque of the SB-SRA started to be generated at a supply pressure of approximately 150 kPa because the initial tension of the extension spring $F_{s}$ was 3.756 N. This preload force was subtracted independently from the pneumatic driving force and was not multiplied by the empirical coefficient α. The output torque showed little variation among the four installation positions of the SB-SRA shown in Figure 10b (max. error: 0.0054 Nm). Here, when the EFPA is assumed to be a typical rigid pneumatic cylinder, the theoretical torque $\tau_{t}$ of the SB-SRA is calculated using $\tau_{t}=2APr$, where A is the cross-sectional area of the EFPA based on its inner diameter of 8 mm, and P is the supply pressure. The theoretical result is approximately 2 Nm when the supply pressure is 500 kPa. On the other hand, the experimental result is approximately 0.51 Nm (500 kPa). This indicates that only a part of the ideal force contributed to the output torque because of friction between the bellows sleeve and the silicone rubber tube, deformation of the silicone rubber tube, and the oblique arrangement of the EFPAs. Therefore, an empirical torque coefficient α was introduced based on the experimental results, as shown in Figure 11. The equation of torque, including the coefficient α based on the above equation is expressed as

(2) $$\tau_{t}=2\alpha APr-F_{s}r_{s}.$$

where $r_{s}$ is the distance from the rotational axis to the point at which the tensile spring force is applied. The factor 2 represents the two EFPAs that are simultaneously pressurized for each rotational direction. The empirical coefficient α, which accounts for the reduction in the effective driving force, was determined to be 0.31 by least-squares fitting of Equation (2) to the measured torque data over the pressure range of 150–500 kPa. Therefore, in future work, the coefficient of α should be investigated in relation to the characteristics of the silicone rubber tube, such as the polymer material, as well as the friction between the tube and the sleeves.

To evaluate the stiffness of the proposed SB-SRA, we investigated the relationship between the bending angle and the bending moment. As a result, the stiffness is very small: 0.0599 Nm/° and 0.0707 Nm/° for the supply pressure of 0 and 500 kPa, respectively.

### 4.3. Dynamic Characteristics of SB-SRA

**Figure 12:**
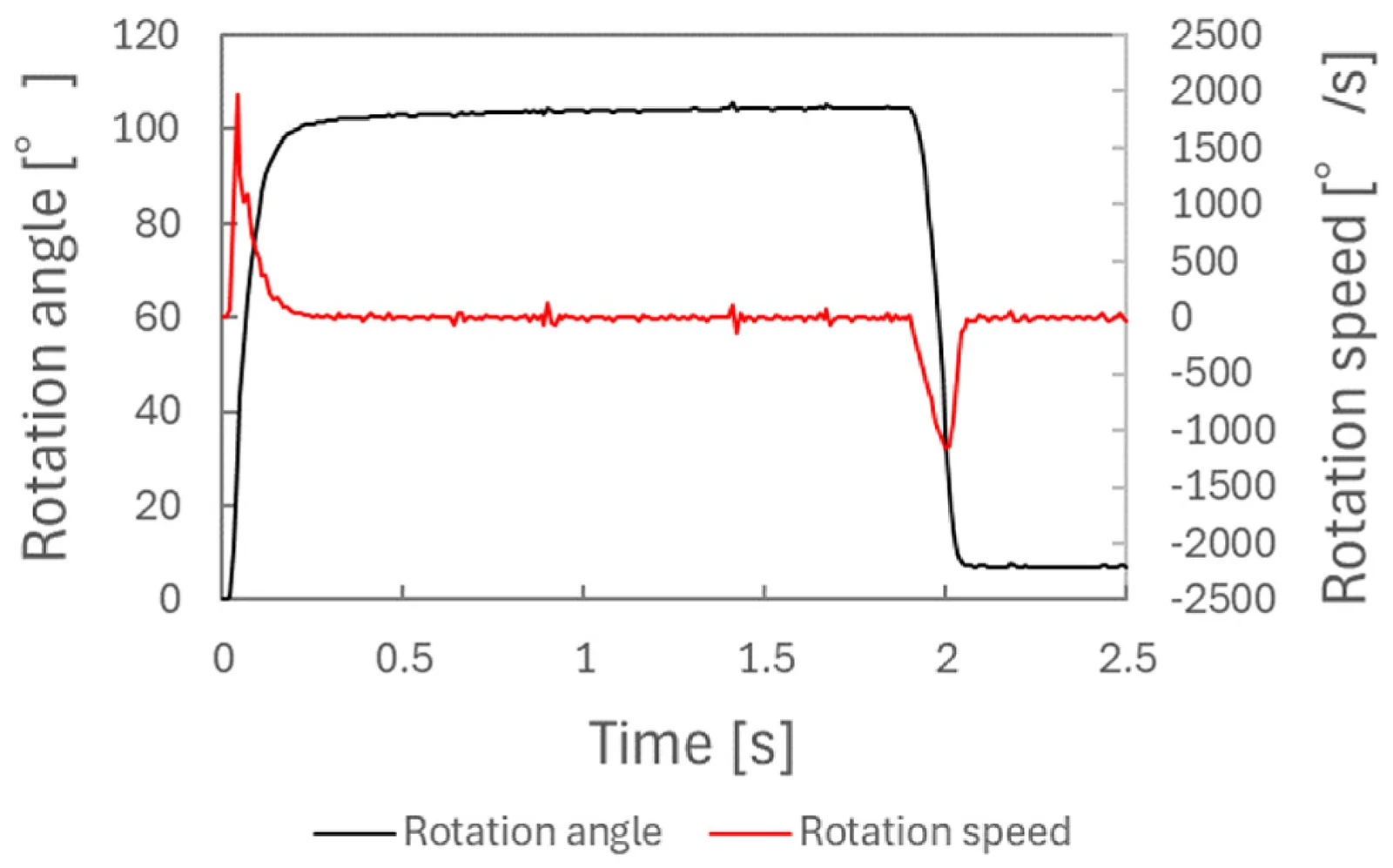
Transient responses of rotation angle and speed of SB-SRA for stepwise supply pressure.

To evaluate the dynamic characteristics of the SB-SRA, a stepwise supply pressure of 500 kPa was applied from the initial state. The transient rotation angle was measured using a rotary potentiometer connected to the rotating shaft of the SB-SRA. The rotational speed was calculated from the change in the measured rotation angle with time. Figure 12 shows the transient responses of the rotation angle and rotational speed.

The dead time and time constant estimated from the step response were approximately 0.02 s and 0.05 s, respectively. Based on the estimated time constant, the −3 dB bandwidth frequency was approximately 3.2 Hz. The maximum rotational speed was approximately 1963.5°/s (327.3 rpm). These results demonstrate the relatively rapid dynamic response of the SB-SRA and provide fundamental dynamic characteristics for its potential application to robotic joints and rehabilitation devices.

## 5. Adjustment of the Hysteresis Characteristic of SB-SRA

**Figure 13:**
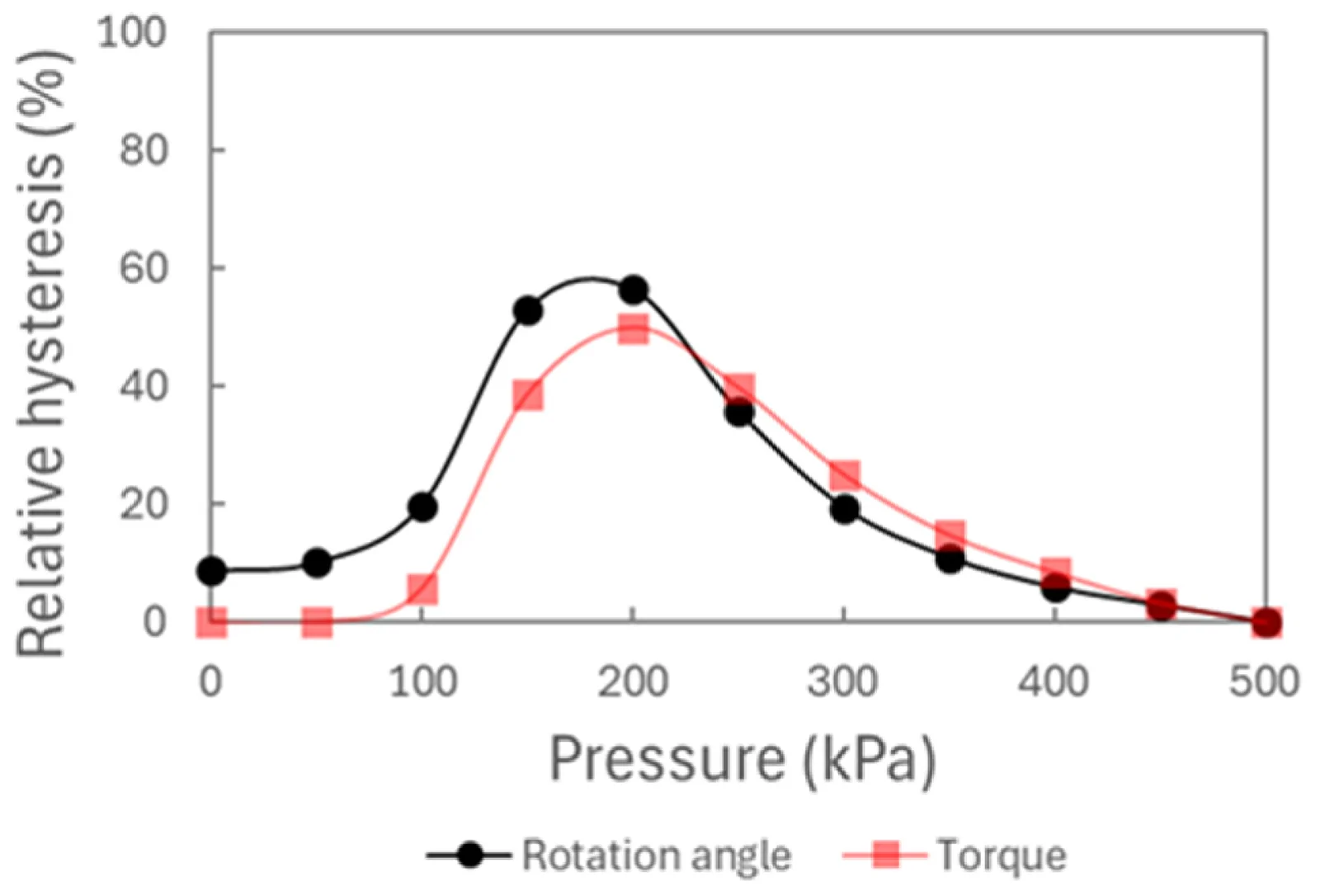
Relationship between pressure and relative hysteresis of rotation angle and torque.

Before describing the compensation method, the hysteresis of the SB-SRA is quantified. Figure 13 shows the relative hysteresis of the rotation angle and the torque as a function of the supply pressure.

Here, the relative hysteresis is defined as the difference between the depressurization and pressurization branches at the same supply pressure, divided by the maximum output obtained in a single rotational direction at 500 kPa, that is, 105° for the rotation angle and 0.51 Nm for the torque; it is not normalized by the total span of the two rotational directions. The hysteresis is largest at 200 kPa for both quantities: the rotation angle was 21.4° during pressurization and 81.3° during depressurization, a difference of 59.9°, corresponding to a relative hysteresis of 57.1%, while the torque difference was 0.252 Nm (49.3%). Above 200 kPa, both generally decrease monotonically, reaching 3.4° (3.2%) and 0.018 Nm (3.5%) at 450 kPa.

**Figure 14:**
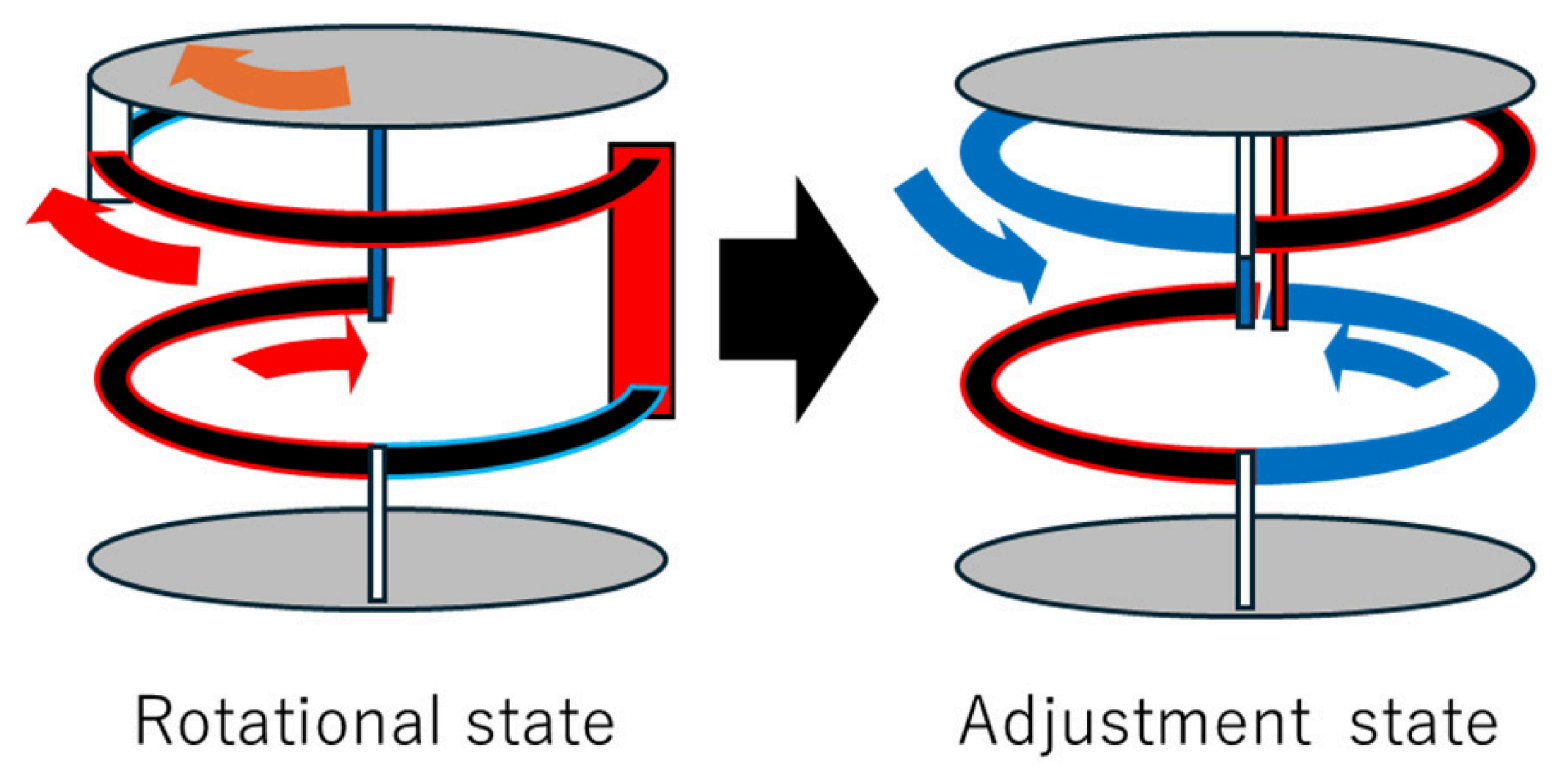
Appearance of hysteresis adjustment by pushing the EFPAs against each other.

According to the mechanism of the SB-SRA shown in Figure 14, both EFPAs corresponding to the two rotational directions can be extended by applying the supply pressure. In other words, by adjusting the displacement of each EFPA, the hysteresis characteristics of the rotation angle and torque of the SB-SRA can be controlled, while maintaining the flexibility, in the same manner as those of a typical pneumatic rotary actuator. In addition, the hysteresis adjustment can be achieved while bending the shaft.

**Figure 15:**
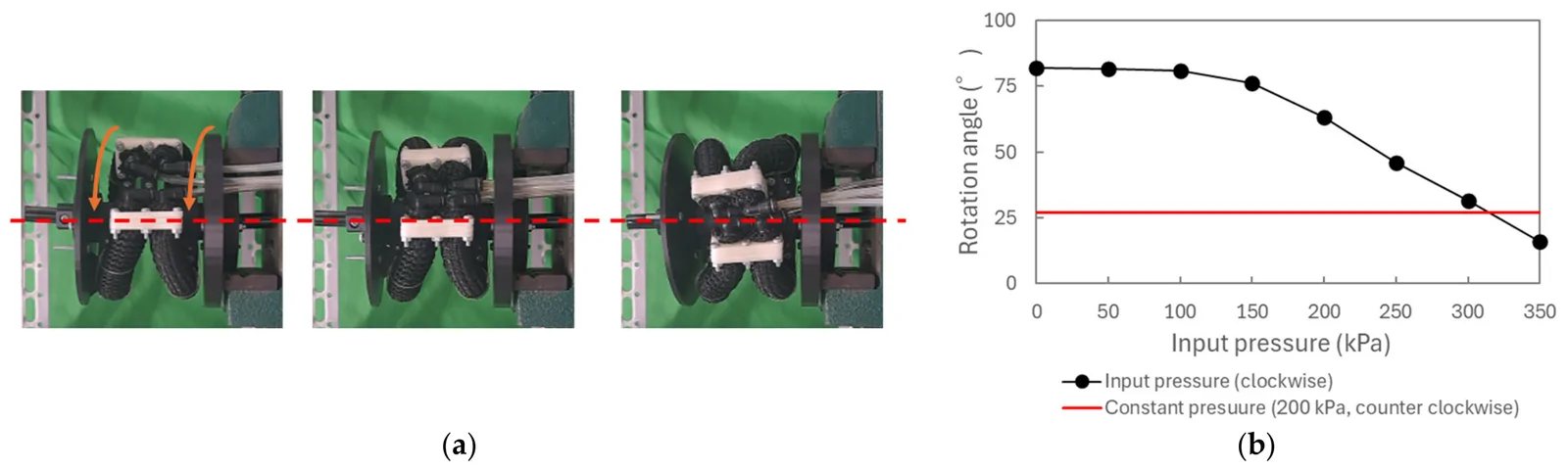
(**a**) Appearance of the opposite rotation direction after the desired angle was reached by the above operation. The antagonized state in which the EFPA for the opposite rotational direction is pushed against the extended EFPA is shown; (**b**) relationship between the input pressure of the EFPA for clockwise rotation and the rotation angle when the inner pressure of the EFPA for counterclockwise rotation was fixed at 200 kPa.

The verification of the hysteresis control method is described below. First, focusing on one rotation direction, the EFPA is pressurized in steps of 100 kPa in supply pressure, and the rotation angle is measured. Next, from the state pressurized to a supply pressure of 500 kPa, the EFPA is decompressed to a desired pressure. The EFPA rotating in the opposite direction is then pressurized and adjusted so that the SB-SRA reaches the rotation angle obtained when pressurized from a pressure of 0, and this pressure is measured. Figure 15a shows the appearance of the opposite rotational direction after the desired angle was reached by the above operation.

Specifically, Figure 15a presents the antagonized state in which the EFPA for the opposite rotational direction is pushed against the extended EFPA, as shown in Figure 14. The shape of the EFPA in the SB-SRA was slightly deformed by pushing the opposite one. The rotation angle was maintained even when the EFPA was bent, as shown in Figure 15a. Therefore, the SB-SRA could achieve adjustment of the rotation angle without breaking the entire structure. Figure 15b shows the relationship between the input pressure of the EFPA for clockwise rotation and the rotation angle. This indicates the change in angle caused by pressurizing the EFPA for clockwise rotation in steps of 50 kPa, while the inner pressure of the EFPA for counterclockwise rotation was fixed at 200 kPa. As the input pressure of the EFPA for clockwise rotation increases, the rotation angle for counterclockwise rotation decreases. When the input pressure becomes approximately 320 kPa, the rotation angle of the SB-SRA agrees with the angle of 27.1° for the supply case shown in Figure 6. Thus, we investigated whether adjusting the opposite EFPA could adjust the hysteresis characteristics even under other supply pressure conditions.

**Figure 16:**
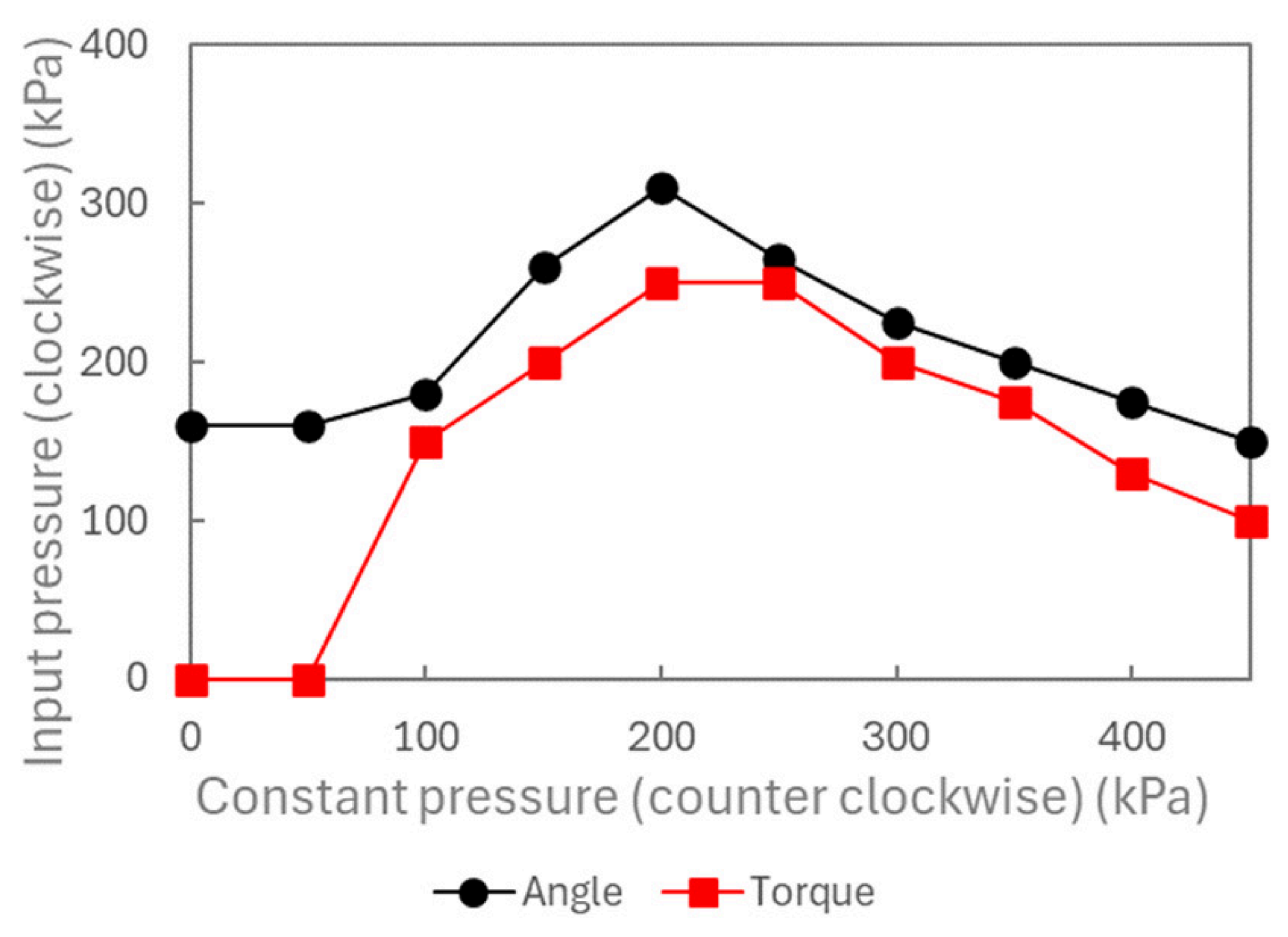
Relationship between the inner pressure of the EFPA for counterclockwise rotation and the input pressure of the EFPA for clockwise rotation required for the hysteresis adjustment of the rotation angle and torque.

**Figure 17:**
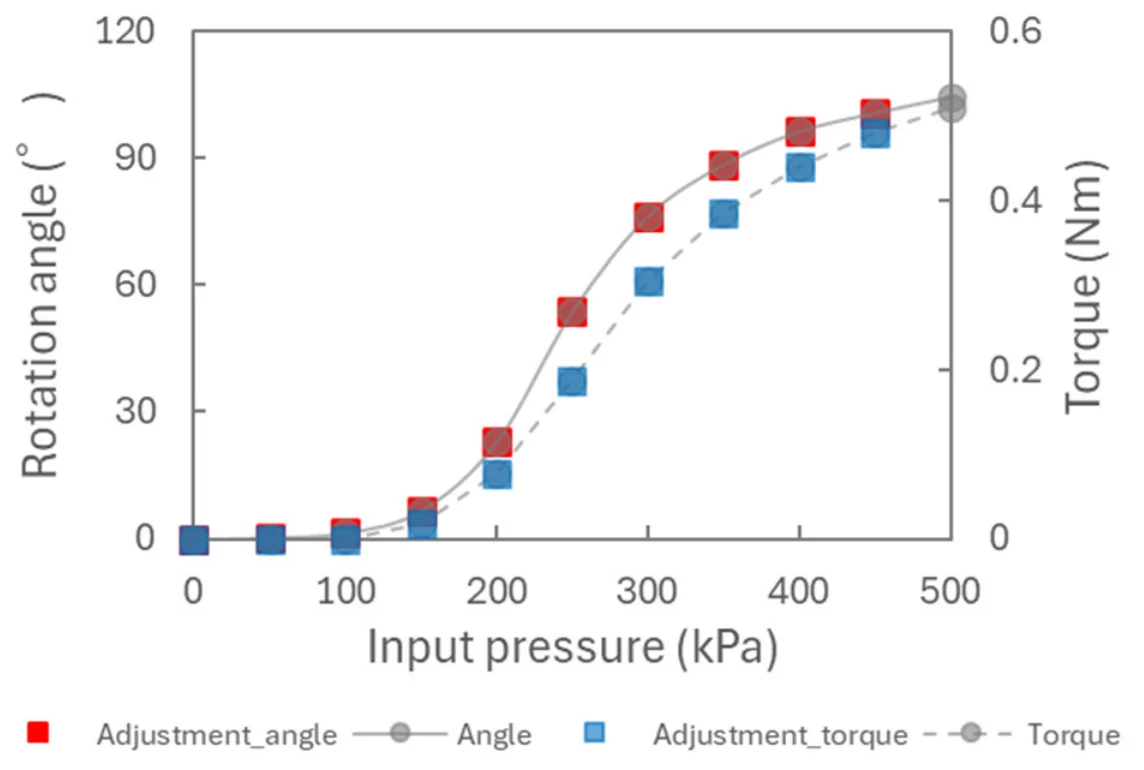
Example of the hysteresis adjustment: rotation-angle and torque characteristics in counter-clockwise rotation.

Figure 16 shows the relationship between the inner pressure of the EFPA to extend in one and opposite directions. In the experiment, in the same manner as described above, the supply pressure applied to the EFPA for clockwise rotation was adjusted so that the rotation angle and torque of the SB-SRA matched the angle obtained when the desired inner pressure was applied to the EFPA for counterclockwise rotation. These results indicate that the larger the hysteresis, the larger the supply pressure required for adjustment. Referring to the hysteresis characteristics shown in Figure 6, the hysteresis at a pressure of 200 kPa is the largest; therefore, the input pressure of the EFPA for clockwise rotation is required the most. As for the torque results, the required input pressure for clockwise rotation is smaller than the result of the rotation angle because the spring pulls it, as shown in Figure 10a. As an example of the hysteresis adjustment, Figure 17 shows the rotation-angle and torque characteristics in counterclockwise rotation obtained by the proposed adjustment method mentioned above. In this procedure, the output rotation angle is adjusted to match the corresponding value on the pressurization branch. The pressure of the opposing EFPA is increased until the measured angle reaches this target value. Therefore, the experiment determines the required adjustment pressure shown in Figure 16, rather than providing an independent measurement of the post-compensation hysteresis. The required pressure was largest at 200 kPa, 310 kPa for the angle and 250 kPa for the torque, which is also the pressure at which the uncompensated hysteresis is largest. This hysteresis adjustment is possible because the proposed SB-SRA has two sets of EFPAs for opposite rotational directions. It is considered that the hysteresis is adjusted because an external force in the contraction direction is applied to the rubber, whose contraction is hindered by friction during decompression. In future work, a force control method should be developed to achieve the desired torque by pressure value.

The adjustment described above is an open-loop, static compensation carried out under quasi-static conditions, in which the supply pressure of the opposing EFPA is set so that the actuator reaches a target angle. It is neither closed-loop feedback control nor a general hysteresis cancelation method valid for arbitrary trajectories. Closed-loop angle and torque control will require a built-in displacement sensor and a dynamic model that accounts for friction between the silicone rubber tube and the bellows sleeve.

## 6. Conclusions

In this study, we proposed a soft rotary actuator with bidirectional rotational motion, approximately symmetric output characteristics, and static hysteresis adjustment, while maintaining a flexible rotational axis.

It was confirmed that the SB-SRA with the proposed mechanism achieves bidirectional rotational motion without interference between the EFPAs. In addition, the actuator maintained bidirectional rotational motion with the flexible shaft bent at 20°.

We investigated the characteristics of the SB-SRA, such as the rotation angle and torque, to verify whether they are symmetrical. As a result, it was confirmed that both the rotation-angle and torque characteristics were approximately symmetric in both rotational directions. The maximum directional differences were 3.3% for the rotation angle and 2.4% for the torque. The maximum rotation angle and torque were approximately 105° and 0.51 Nm, respectively, at a supply pressure of 500 kPa.

Static hysteresis adjustment was achieved by applying pressure to the opposing EFPAs without using additional braking components. The maximum relative hysteresis before adjustment was 57.1% for the rotation angle and 49.3% for the torque at 200 kPa. At this pressure, the opposing pressure required for the adjustment was 310 kPa for the rotation angle and 250 kPa for the torque. These results demonstrate that the proposed SB-SRA can provide approximately symmetric bidirectional rotation and static hysteresis adjustment while retaining the flexibility of the rotational axis.

As future work, the SB-SRA will be integrated with a built-in displacement sensor and a closed-loop controller for accurate position control, and its performance will be evaluated in practical robotic applications requiring rotational motion. The present study employed simplified quasi-static models to estimate the fundamental rotation-angle and torque characteristics of the SB-SRA. A detailed numerical model would need to account for the nonlinear large deformation of the silicone rubber tube, deformation of the bellows sleeve, and contact and friction between these components. As future work, these effects will be incorporated into a numerical model to improve the prediction of the rotation angle, output torque, and hysteresis characteristics and to support the control and design of the actuator.

In addition, an analytical model including the friction between the silicone rubber tube and the bellows sleeve will also be proposed to predict and compensate for the hysteresis and to improve the control performance of the actuator.

## Figures and Tables

**Table 1:** Comparison of the proposed SB-SRA with representative soft rotary actuators.

	Proposed Soft Actuator	[12] Yan 2018	[25] Sanan 2014	[23] Li 2023	[26] Liu 2021
Actuation principle	Extension in both rotational directions	Inflation of spiral chambers	Inflation of opposed fabric helices	Folding of origami shell	Deformation of curved PAMs
Size (φ × L)	110 × 90 mm	18 × 50 mm	Not reported	Not reported	110 × 180 mm
Weight	0.5 kg	Not reported	0.01 kg	Not reported	1.5 kg
Bidirectional rotation	○	×	○ (binary only)	×	○
Symmetric output	○	×	×	×	○
Flexibility	○	○	○	○	×
Bendable rotational axis	○ (20°)	×	×	×	×
Max. angle/torque	±105°/ 0.51 Nm	110°/ 0.026 Nm	40°/ Not measured	435°/ 0.024 Nm	±68.5°/ 4.76 Nm

○ Satisfied; × Not satisfied or not reported. Angles and torques are experimentally obtained values. Those of the proposed SB-SRA are presented in Section 4. “Binary only” indicates actuation limited to two discrete states [18].

## Data Availability

The data presented in this study are available on request from the corresponding author.
